# Estimation of the *In Vivo* MIC of Cipargamin in Uncomplicated Plasmodium falciparum Malaria

**DOI:** 10.1128/AAC.01940-16

**Published:** 2017-01-24

**Authors:** Tran Tinh Hien, Nicholas J. White, Nguyen Thanh Thuy-Nhien, Nhu Thi Hoa, Phung Duc Thuan, Joel Tarning, François Nosten, Baldur Magnusson, Jay Prakash Jain, Kamal Hamed

**Affiliations:** a Oxford University Clinical Research Unit-Hospital for Tropical Diseases, Ho Chi Minh City, Vietnam; b Mahidol-Oxford Tropical Medicine Research Unit, Faculty of Tropical Medicine, Mahidol University, Bangkok, Thailand; c Shoklo Malaria Research Unit, Faculty of Tropical Medicine, Mahidol University, Mae Sot, Thailand; d Centre for Tropical Medicine and Global Health, Nuffield Department of Clinical Medicine, University of Oxford, Oxford, United Kingdom; e Novartis Pharma AG, Basel, Switzerland; f Novartis Healthcare Pvt. Ltd., Hyderabad, India; g Novartis Pharmaceuticals Corporation, East Hanover, New Jersey, USA

**Keywords:** malaria, resistance, cipargamin, pharmacokinetic-pharmacodynamic model, clinical trial

## Abstract

The MIC of an antimalarial drug for a particular infection is the drug level associated with a net parasite multiplication rate of one per asexual cycle. To ensure the cure of malaria, the MIC must be exceeded until all parasites have been eliminated. The development of highly sensitive and accurate PCR quantitation of low-density malaria parasitemia enables the prospective pharmacokinetic-pharmacodynamic (PK-PD) characterization of antimalarial drug effects and now allows identification of the *in vivo* MIC. An adaptive design and a PK-PD modeling approach were used to determine prospectively the MIC of the new antimalarial cipargamin (KAE609) in adults with uncomplicated Plasmodium falciparum malaria in an open-label, dose-ranging phase 2a study. Vietnamese adults with acute P. falciparum malaria were allocated sequentially to treatment with a single 30-mg (*n* = 6), 20-mg (*n* = 5), 10-mg (*n* = 7), or 15-mg (*n* = 7) dose of cipargamin. Artemisinin-based combination therapy was given after parasite densities had fallen and then risen as cipargamin levels declined below the MIC but before a return of signs or symptoms. The rates of parasite clearance were dose dependent, with near saturation of the effect being seen at an adult dose of 30 mg. The developed PK-PD model accurately predicted the therapeutic responses in 23/25 patients. The predicted median *in vivo* MIC was 0.126 ng/ml (range, 0.038 to 0.803 ng/ml). Pharmacometric characterization of the relationship between antimalarial drug concentrations and parasite clearance rates following graded subtherapeutic antimalarial drug dosing is safe and provides a rational framework for dose finding in antimalarial drug development. (This study has been registered at ClinicalTrials.gov under identifier NCT01836458.)

## INTRODUCTION

The emergence of resistance to current antimalarial drugs threatens to reverse recent substantial gains in the control and elimination of malaria. Several factors contribute to the emergence and spread of resistance, notably, uncontrolled use of monotherapies, poor-quality medicines, and systematic underdosing (1, 2). Therefore, maximizing the lifetime of any new antimalarial drug depends not only on the propensity for resistance to arise or for a drug to share cross-resistance with existing drug classes but also on finding the best treatment dose during drug development (2).

An alternative to previous, largely empirical approaches to dose finding for new antimalarial drugs is to determine prospectively, *in vivo*, the MIC (2). The MIC has been defined to be the plasma or blood drug concentration associated with a parasite multiplication rate of one per cycle and therefore represents a therapeutic target that must be exceeded for a sufficient duration to guarantee a cure (3). In recent years, *ex vivo* experimental systems, animal models, and, more recently, human challenge models have provided valuable information on antimalarial pharmacokinetic (PK)-pharmacodynamic (PD) relationships, although their direct relevance to the treatment of symptomatic malaria remains uncertain (4). Ideally, the key PK-PD determinants of the therapeutic response should be determined in the natural disease.

The spiroindolone cipargamin (formerly KAE609) is a new antimalarial drug with potent activity against Plasmodium falciparum and Plasmodium vivax (5–7). Cipargamin inhibits the P-type cation-translocating ATPase ATP4 of P. falciparum (PfATP4), disturbing parasite sodium homeostasis and causing osmotic dysregulation (5, 8, 9). This novel mechanism of action affecting all stages of intraerythrocytic parasite development probably explains why cipargamin treatment causes extremely rapid parasite clearance (5, 6, 10).

In the first study of its kind, we aimed to estimate prospectively the *in vivo* MIC of cipargamin in acute uncomplicated P. falciparum malaria. This was made possible by the development of sensitive and accurate quantitative PCR (qPCR) measurement of parasite nucleic acids, which permits tracking of parasitemia at densities well below those causing illness and, thus, the characterization of recrudescence without discomfort or risk for the patient (11–13).

## RESULTS

### Patients.

Twenty-five adult Vietnamese male patients with acute uncomplicated falciparum malaria were enrolled between 15 January 2014 and 12 March 2015. The first three groups received cipargamin in descending dose order: 30 mg (*n* = 6), 20 mg (*n* = 5), and 10 mg (*n* = 7) (Table 1). One patient in the 30-mg group received 21 mg in error and was included in the 20-mg group for the analysis. Because of the inadequate therapeutic responses in the 10-mg group, dosing of the fourth group (*n* = 7) was increased to 15 mg. Sixteen patients received early rescue treatment (30 mg, *n* = 3/6; 20 mg, *n* = 2/5; 10 mg, *n* = 5/7; 15 mg, *n* = 6/7), four because of early treatment failure (ETF) and 12 because of later recrudescence (see Fig. S1 in the supplemental material).

**TABLE 1:** Patient demographics and baseline characteristics by cipargamin dose

Characteristic	Dose 1 (30 mg, *n* = 6)	Dose 2 (20 mg, *n* = 5^*a*^)	Dose 3 (10 mg, *n* = 7)	Dose 4 (15 mg, *n* = 7)	Total (*n* = 25)
Age (yr)					
Mean (SD)	33.2 (12.91)	32.2 (6.98)	30.6 (8.24)	34.7 (8.79)	32.7 (9.04)
Median (range)	33.5 (20–52)	30.0 (23–41)	30.0 (20–42)	35.0 (20–46)	31.0 (20–52)
No. (%) male subjects	6 (100)	5 (100)	7 (100)	7 (100)	25 (100)
No. (%) Asian subjects	6 (100)	5 (100)	7 (100)	7 (100)	25 (100)
Ht (cm)					
Mean (SD)	168.8 (5.49)	163.8 (5.36)	163.7 (8.30)	161.9 (2.41)	164.4 (6.06)
Median (range)	167.5 (163–176)	162.0 (157–171)	162.0 (150–175)	161.0 (159–165)	164.0 (150–176)
Wt (kg)					
Mean (SD)	60.7 (7.45)	56.8 (3.35)	60.8 (11.41)	56.9 (5.44)	58.9 (7.56)
Median (range)	63.5 (50–69)	57.0 (53–62)	60.0 (42–78)	59.0 (50–65)	59.0 (42–78)
Body mass index (kg/m^2^)					
Mean (SD)	21.3 (2.65)	21.2 (1.16)	22.5 (2.42)	21.7 (2.07)	21.7 (2.12)
Median (range)	20.8 (18.8–25.7)	21.0 (20.2–23.1)	22.9 (18.7–26.4)	23.0 (18.6–23.9)	21.2 (18.6–26.4)

a One patient in the 30-mg cohort received 21 mg in error (a 1-mg capsule was mistakenly administered in place of the required 10-mg capsule). Data for this patient were analyzed with those for the 20-mg cohort for all analyses.

### Therapeutic responses.

**FIG 1:**
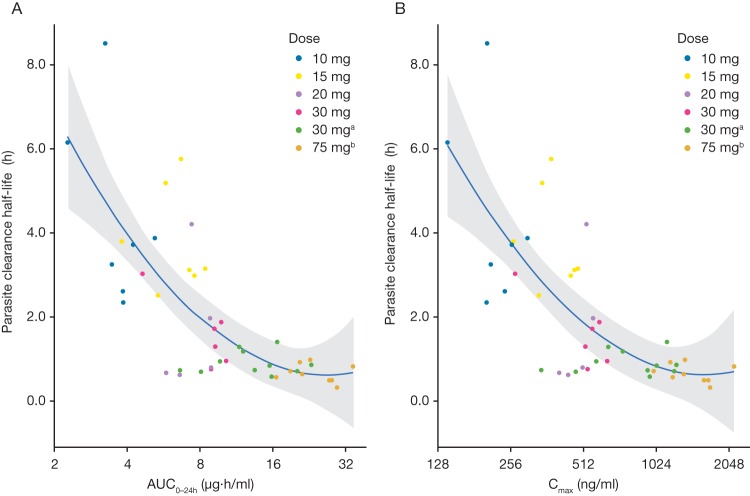
Parasite clearance half-life versus AUC_0–24_ (A) and parasite clearance half-life versus *C*_max_ (B). Circles represent individual AUCs, the different colors represent the corresponding cipargamin doses, and the blue line and shaded area represent LOESS smooth with the uncertainty band. ^a^, data are from study X2201 (5); ^b^, data are from study X2202 (registered at ClinicalTrials.gov under identifier NCT01860989). AUC_0–24_, area under the plasma concentration-time curve from time zero to 24 h postdosing; C_max_, maximum plasma concentration.

Rates of fever and parasite clearance were approximately dose dependent (Table S1). Mean ± standard deviation (SD) parasite clearance half-life (PC_1/2_) estimates were 4.35 ± 2.21, 3.79 ± 1.22, 1.91 ± 1.64, and 1.47 ± 0.83 h for doses of 10, 15, 20, and 30 mg, respectively. An asymptotic nonlinear model described the relationship between PC_1/2_ and both the area under the plasma concentration-time curve (AUC) between 0 and 24 h (AUC_0–24_) and the observed maximum plasma concentration (*C*_max_) well and suggested saturation of the effect at higher doses of cipargamin (Fig. 1). Despite trends for an increased 28-day PCR-corrected cure rate with higher doses and increased exposure (AUC) (Table S1), no individual PK parameter predicted a cure (data not shown).

### Safety.

Nineteen (76%) patients reported at least one adverse event (AE) (Table S2). Three AEs in the 30-mg group were considered related to the study treatment (nausea, myalgia, and headache). There were no serious AEs or AEs leading to study discontinuation. Most laboratory abnormalities did not exceed grade 2 severity; six patients had grade 3 abnormalities. One patient with a history of hypertension who received 30 mg had a rise in blood pressure to 160/100 mm Hg that resolved after treatment with furosemide at 20 mg.

### *In vitro* susceptibility to cipargamin.

Population estimates for the slope and 50% inhibitory concentration (IC_50_) were −1.22 (95% confidence interval [CI], −1.35 to −1.09) and 2.97 ng/ml (95% CI, 2.70 to 3.26 ng/ml), respectively, with the median individual estimated IC_50_ being 2.38 ng/ml (range, 0.468 to 17.2 ng/ml; interquartile range [IQR], 1.25 to 4.07 ng/ml).

### Pharmacokinetic modeling.

**FIG 2:**
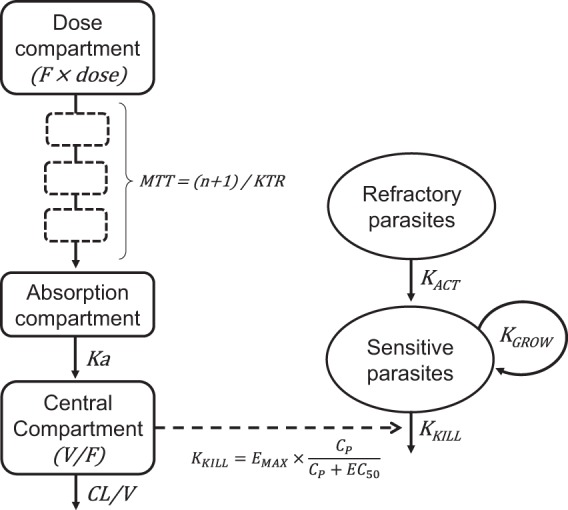
Schematic representation of the final model describing cipargamin pharmacokinetics and pharmacodynamics in patients with uncomplicated P. falciparum malaria. The fraction of asexual parasites that were fully drug sensitive was estimated at enrollment, and the observed total parasitemia is the sum of sensitive and refractory parasites. CL/*V*, apparent elimination clearance; *C_p_*, cipargamin plasma concentration; EC_50_, concentration needed for 50% of the maximum effect; *E*_max_, maximum effect (maximum parasite reduction rate; this effect is dose dependent, as specified in Table 3); *F*, relative oral bioavailability; *K_a_*, first-order absorption rate constant from the last transit compartment to the central compartment; *K*_act_, first-order rate constant for refractory parasites to become active; *K*_grow_, parasite multiplication rate fixed to 10-fold multiplication per 48-h cycle; *K*_kill_, first-order rate constant for cipargamin-dependent parasite killing; KTR, first-order transit rate constant; MTT, mean transit time through the transit compartments; *n*, number of transit compartments; *V*/*F*, apparent volume of distribution.

The PK properties of cipargamin were well described by a flexible transit-absorption model followed by a one-compartment disposition model (Fig. 2). Allowing interindividual variability on the relative bioavailability significantly improved the model fit. Body weight was incorporated as a fixed allometric function on the clearance and volume parameters. Dose did not significantly affect the PK properties, suggesting dose-linear kinetics (Tables 2 and 3 and Fig. S2). The final PK model (Table 3) showed excellent overall diagnostic performance (Fig. S3 and S4).

**TABLE 2:** Summary of individual PK parameter estimates from the final model describing cipargamin PKs in patients with uncomplicated P. falciparum malaria^*b*^

Parameter^*a*^	Result for the following dose:			
	30 mg (*n* = 6)	20 mg (*n* = 5)	15 mg (*n* = 7)	10 mg (*n* = 7)
*C*_max_ (ng/ml)	597 (248–687)	535 (434–678)	442 (312–618)	247 (155–413)
*T*_max_ (h)	3.51 (2–10)	3.00 (2–4)	3.00 (2–4)	4.02 (2–6)
AUC_inf_ (μg · h/ml)	16.7 (7.75–18.80)	10.1 (8.30–15.60)	11.3 (5.28–14.40)	6.4 (3.87–9.10)
*t*_1/2_ (h)	16.7 (12.2–29.2)	15.4 (12.0–16.2)	14.9 (11.4–28.8)	17.3 (15.6–25.0)

a Individual *post hoc* empirical Bayes estimates from the final population pharmacokinetic (PK) model.

b All values are reported as median (range). *C*_max_, maximum plasma concentration; *T*_max_, time to reach *C*_max_; AUC_inf_, accumulated area under the plasma concentration-time curve from time zero to infinity; *t*_1/2_, terminal elimination half-life.

**TABLE 3:** Population parameter estimates of the final model describing cipargamin PKs and PDs in patients with uncomplicated P. falciparum malaria^*c*^

Model and parameter	Population estimate^*a*^ (% RSE^*b*^)	95% CI^*b*^	% CV for IIV^*a*^ (% RSE^*b*^)	95% CI^*b*^
PK model				
CL/*F* (liters/h)	1.72 (5.69)	1.55–1.94	18.5 (12.4)	13.5–21.8
*V/F* (liters)	40.6 (4.71)	37.3 −44.7		
No. trans comp	3 *fix*			
MTT (h)	0.867 (12.6)	0.682–1.11	65.2 (13.5)	47.5–79.9
*K_a_* (h^−1^)	1.65 (25.2)	1.04–2.81	176 (30.4)	107–312
*F* (%)	100 *fix*		26.2 (25.3)	13.6–37.6
σ (% CV)	17.5 (10.3)	14.3–21.1		
PD model				
*K*_grow_ (h^−1^)	0.0479 *fix*			
*E*_max_ (h^−1^)	0.564 (12.9)	0.383–0.710	62.2 (33.6)	45.1–90.2
EC_50_ (ng/ml)	0.354 (16.7)	0.222–0.466		
*F*_sen_ (%)	99.1 (0.132)	98.8–99.4	81.8 (39.2)	58.9–194
*K*_act_ (h^−1^)	0.0987 (14.7)	0.0592–0.123	41.5 (42.0)	30.2–67.7
COV_dose_*E*_max__	0.0463 (14.9)	0.0318–0.0604		
σ (% CV)	109 (17.0)	94.8–178		

a Population mean values and interindividual variability (IIV) were estimated by the use of NONMEM software. The coefficient of variation (CV) for interindividual variability was calculated as $100\times \sqrt{{\text{e}}^{\text{estimate}}-1}$. Population mean parameter estimates were calculated for a typical patient with a body weight of 59 kg receiving a study drug dose of 10 mg.

b Relative standard error (RSE) was calculated as 100 × (standard deviation/mean parameter estimate) from 1,000 and 500 successful iterations for the PK and PK-PD models, respectively, of a nonparametric bootstrap diagnostic. The 95% confidence interval (CI) was characterized as the 2.5th to 97.5th percentiles of the bootstrap estimates.

c CL/*F*, apparent elimination clearance; *V/F*, apparent volume of distribution; No. trans comp, number of transit compartments in the absorption model; MTT, mean transit time through the transit compartments; *K_a_*, first-order absorption rate constant from the last transit compartment to the central compartment; *F*, relative oral bioavailability; σ, unexplained residual error; *K*_grow_, parasite multiplication rate fixed to 10-fold multiplication per 48-h cycle; *E*_max_, maximum effect (maximum parasite reduction rate); EC_50_, concentration needed for 50% of the maximum effect; *F*_sen_, fraction of total asexual parasites which were fully drug sensitive; *K*_act_, first-order rate constant for refractory parasites to become active; COV_dose_*E*max_, exponent of the relationship between dose and *E*_max_ [i.e., θ_i_ = θ_TVE_max__ × (dose/10)^COV_dose_*E*max_^] (see Equation 7 in the supplemental text); PD, pharmacodynamic; PK, pharmacokinetic.

### Pharmacodynamic modeling.

**FIG 3:**
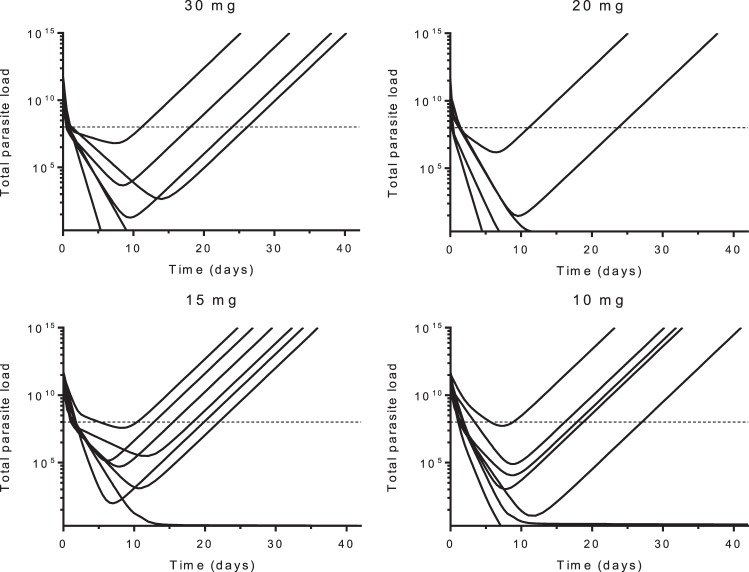
Individually predicted parasite clearance curves, using the final model describing cipargamin pharmacokinetics and pharmacodynamics in patients with uncomplicated P. falciparum malaria. Each solid line represents the predicted total parasite load after dosing over time for an individual patient. The broken horizontal lines indicate the level of detection by microscopy.

As individual patient malaria parasite multiplication rates (*K*_grow_) before drug administration are unknown, *K*_grow_ was fixed to 10 per life cycle (i.e., 48 h) (14, 15). The initial implementation of the PK-PD model assumed a homogeneous parasite population and first-order drug-dependent parasite removal (first-order rate constant for cipargamin-dependent parasite killing [*K*_kill_]). However, individual and population parasite clearance curves showed a clearly biphasic parasitemia decline that could not be adequately explained by gametocytemia. A small, refractory parasite subpopulation was considered the most likely explanation, and its incorporation significantly improved the model fit. The fraction of all asexual parasites that were refractory (1 − *F*_sen_, where *F*_sen_ is the fraction of total asexual parasites which were fully drug sensitive) was estimated, but interindividual variability was allowed. Their activation (awakening; first-order rate constant for refractory parasites to become active [*K*_act_]) and then removal was also estimated to explain cures. Higher doses and correspondingly higher plasma concentrations were associated with significantly faster maximum parasite killing rates (the maximum effect [*E*_max_; maximum parasite reduction rate]). The final PK-PD model (Table 3) showed adequate overall diagnostic performance (Fig. S3 and S4) and characterized therapeutic responses correctly for 23 of the 25 patients (i.e., cure, ETF, or later recrudescence; Fig. 3). The estimated median *in vivo* MIC was 0.126 ng/ml (range, 0.0375 to 0.803 ng/ml; IQR, 0.0786 to 0.273 ng/ml), occurring at a median interval of 7.45 days (range, 5.29 to 11.4 days; IQR, 6.51 to 9.32 days) after drug administration for patients correctly predicted as having recrudescent infections (*n* = 12). Time to MIC is a function of dose, as lower doses and, subsequently, lower drug concentrations reach MIC values more rapidly than higher doses, assuming similar PKs and parasite characteristics. Patients correctly predicted to be cured (*n* = 7) showed a maximum median MIC value of 0.236 ng/ml (range, 0.03 to 6.33 ng/ml; IQR, 0.08 to 1.47 ng/ml), defined as the cipargamin concentration when the predicted parasite numbers fell below 10.

## DISCUSSION

Antimalarial drugs are deployed in combinations both to optimize efficacy and to prevent or delay the emergence of resistance (http://www.who.int/malaria/publications/atoz/9789241549127/en/). Fixed-dose combinations (FDCs) are needed to ensure adherence to both components and prevent the use of monotherapies that could select for resistance to individual drugs. It is essential that the optimum dose of a newly developed compound be chosen before the drug is tied into a FDC. While patients with partial immunity may clear partially treated infections, to ensure a cure with the individual drug component in all patients, plasma concentrations must exceed the MIC for a sufficient time to eliminate all the blood-stage parasites. Identifying the dose and dosing strategy that achieves this requires characterization of two independent variables: the population PK properties of the antimalarial drugs in the target populations and the *in vivo* drug susceptibility of the infecting malaria parasite populations. The MIC can be estimated accurately *in vivo* only if there is recrudescence. As the MIC depends both on host factors, notably, immunity, and on the susceptibility of the infecting parasites, it is best assessed in patients from areas with low levels of malaria parasite transmission and, consequently, in patients with low levels of background immunity where multidrug-resistant parasites are prevalent, such as the patients evaluated in the present study. The final dosage in a combination regimen is determined by the predicted duration for which the MIC is exceeded in all relevant patient groups (on the basis of population PK modeling); global variations in malaria parasite susceptibility; practical considerations, such as weight banding; any interactions with the partner drugs; and safety and tolerability (2). This first prospective determination of the *in vivo* MIC in a dose-finding study was made possible by the recent development of sensitive PCR quantitation methods. The MIC of cipargamin was determined safely without discomfort to the patients, all of whom received curative treatment before any signs or symptoms developed.

In this study, the PK properties of the novel spiroindolone antimalarial cipargamin were described satisfactorily by a one-compartment disposition model with flexible absorption and were similar to results from previous studies in malaria patients (5). Exposure to cipargamin was approximately dose proportional, as demonstrated previously in human volunteers (10). In the first exploratory studies conducted in Thai patients with P. falciparum or P. vivax malaria, cipargamin cleared the parasitemia more rapidly than any other known antimalarial drug (5). In the current study, parasite clearance rates increased with increasing cipargamin exposure, approaching a plateau with the 30-mg dose. The PK-PD modeling predicted MIC values ranging from 0.0375 to 0.803 ng/ml (median, 0.126 ng/ml). *In vitro* susceptibility was assessed by the trophozoite maturation test, which measures ring-stage susceptibility, the main determinant of parasite clearance. In the current study, by conventional 48-h *in vitro* tests, which assess the full asexual cycle, mean IC_50_s ranged from 0.195 to 0.546 ng/ml for laboratory isolates, and all IC_50_s were below 3.9 ng/ml for fresh isolates from malaria patients. The results of this study suggest that the *in vivo* MIC for cipargamin is in a concentration range associated with approximately 10% inhibition of trophozoite development and 20 to 50% inhibition of schizont maturation in *ex vivo* systems (although the milieu and, notably, protein binding are different between the two).

In murine models, in which two-compartment disposition models were fitted, cipargamin AUC was a better determinant of parasite clearance than *C*_max_ after single doses (16). In this clinical study, a clear dose-response relationship was evident and a single-compartment model fitted the concentration data satisfactorily. So, *C*_max_ and AUC were closely related, and there was no clear superiority of one over the other as a predictor of the parasitological response.

There are several limitations to PK-PD modeling based upon quantitative DNA estimates. First, DNA measurements do not distinguish between asexual- and sexual-stage parasites or between quiescent and dividing parasites. However, the drug effect being assessed is the effect on dividing asexual-stage parasites and in the case of cipargamin reflects the splenic clearance of red blood cells containing damaged, osmotically dysregulated parasites (17). As parasite densities decline, the more slowly cleared sexual-stage parasites and any drug-refractory forms comprise an increasing proportion of the DNA signal. Recent *ex vivo* studies suggest that cipargamin does not induce the same refractory forms observed with artemisinin drugs (18), so the nature of the parasite forms, which persist despite exposure to high cipargamin concentrations, clearly requires further study (18, 19). Low-dose primaquine was not given as a rapid gametocytocide in this study because cipargamin was considered to have gametocytocidal activity, but this was insufficient. Thus, in some cases, as a result of both drug-refractory parasite stages and persistent gametocytemia, the curve for the fall and subsequent rise in the observed DNA signal was flat bottomed rather than parabolic. Second, while ultrasensitive PCR is 1,000 times more sensitive than microscopy and can detect parasites down to densities of ∼20/ml, parasite densities at the MIC were sometimes lower than this detection threshold; in both cases, the MIC had to be extrapolated rather than observed. Detection of mRNA is potentially more sensitive than detection of DNA, as there are potentially thousands of mRNA transcripts per cell, but their numbers vary with time, making accurate parasite quantitation potentially difficult. However, changing values are useful as indicators of trend and so informed the DNA-based assessments. For example, rising parasite DNA levels at a time of rising numbers of ring-stage mRNA transcripts and falling gametocyte mRNA levels unequivocally identified parasite multiplication.

In conclusion, the rates of P. falciparum parasite clearance were dose dependent, with near saturation of the maximum effect occurring at a cipargamin dose of 30 mg. The developed PK-PD model predicted the therapeutic responses accurately in the majority of falciparum malaria patients.

## MATERIALS AND METHODS

### Patients.

Adult Vietnamese patients who were aged 20 to 60 years, who weighed between 40 and 90 kg, and who had acute P. falciparum monoinfection, as confirmed by microscopy (asexual parasite count 5,000 to 50,000/μl), were recruited if they could take oral medications reliably and if their axillary temperature or oral, tympanic, or rectal temperature was higher than ≥37.5°C or ≥38°C, respectively, at screening or during the previous 24 h. The patients gave fully informed written consent to the study procedures. Exclusion criteria included signs or symptoms of severe malaria, mixed Plasmodium infection, and use of an antimalarial drug within 2 months (see the supplemental text).

### Study design.

An adaptive single-dose de-escalation design was employed. The study (which is registered at ClinicalTrials.gov under identifier NCT01836458) was performed at a single center in Vietnam in accordance with the ethical principles of the Declaration of Helsinki. The study protocol was approved by the ethics committee of the Vietnamese Ministry of Health, the Oxford Tropical Research Ethics Committee (OxTREC), and the study center's institutional review board.

Evaluations of six cipargamin doses running in descending sequence (30, 20, 10, 5, 2, and 1 mg) until the MIC was identified were planned, and approximately eight patients were to be enrolled per treatment group. The option of dose adjustment on the basis of data arising during the study was available. The 30-mg adult dose has previously been shown to clear parasitemia (as assessed by microscopy) in less than 24 h in both P. falciparum and P. vivax malaria (5). During and after each stratum, the data-monitoring committee decided whether to reduce, maintain, or increase the dose. Cipargamin capsules were supplied at doses of 1, 10, and 25 mg and were administered orally as a single dose under direct observation (day 1).

In order to estimate the *in vivo* MIC safely, it is necessary for parasitemia to fall below the level of detection by microscopy and then rise again as drug levels decline below the MIC. The antimalarial plasma concentration at the vertex of this approximate parabola is considered the MIC (2). Antimalarial treatment was given when there was an unequivocal rise in parasite densities indicating recrudescence but before a return of signs or symptoms (see the supplemental text). Thus, the national standard antimalarial treatment course of dihydroartemisinin-piperaquine (DHA-PPQ; 40 mg and 320 mg per tablet, respectively [Arterakine]), in which four, two, and two tablets are administered on days 1, 2, and 3, respectively, and primaquine, in which four 7.5-mg tablets are administered as a single dose, was given to all patients on day 42 or earlier in the case of ETF or rising parasitemia, as determined by qPCR. ETF was defined as clinical deterioration or a lack of clinical/parasitemia improvement at 24 h, parasitemia of >75,000/μl at or after 12 h postdosing, any parasitemia detected by microscopy with fever (at ≥48 h postdosing), or parasitemia of >100/μl on the basis of microscopy without fever (at ≥72 h postdosing).

### Study assessments and procedures.

Vital signs were recorded and blood samples were taken every 4 to 6 h until defervescence and two consecutive negative microscopy parasite counts, then once daily until day 8, then on alternate days until day 28, and on days 35 and 42. Serial parasite densities were measured from thick and thin blood film examinations at higher densities and then by a validated real-time qPCR of the P. falciparum 18S rRNA gene (limit of detection, 22 parasites/ml) conducted in real time on-site and were reported daily to the clinical investigators (20). Samples for ring-stage and gametocyte (P. falciparum s25 [Pfs25]) mRNA transcripts were also taken and measured at the Queensland Institute of Medical Research (QIMR) Berghofer Medical Research Institute (21). Blood samples (3 ml) for plasma cipargamin concentration measurements were taken at 0 (predose), 0.5, 1, 2, 3, 4, 5, 6, 8, 10, 12, 16, 20, 24, 36, 48, 72, 96, 120, 144, and 168 h after dosing. Samples were stored at −70°C until analysis using a validated liquid chromatography-tandem mass spectrometry assay with a lower limit of quantitation of 1 ng/ml (5, 10). The values for more than 67% of the triplicate quality control samples at five different concentrations were within ±15.0% of the individual values. Safety assessments included monitoring of vital signs and laboratory values, electrocardiography, and adverse events (AEs), classified according to the Common Terminology Criteria for Adverse Events (CTCAE; version 4.03; http://evs.nci.nih.gov/ftp1/CTCAE/CTCAE_4.03_2010-06-14_QuickReference_5x7.pdf).

### *In vitro* susceptibility testing.

The *in vitro* susceptibility of the P. falciparum parasites in pretreatment blood samples to cipargamin was measured using a 24-h trophozoite maturation assay (22). Relative parasite maturation and drug concentrations were fitted in GraphPad Prism (version 6.01) software (GraphPad, La Jolla, CA, USA) using the dose-response package. All response data were fitted simultaneously to estimate the population mean slope of the dose-response curve and the IC_50_ and to characterize variability. All individual isolate data were fitted separately using the population slope estimate to generate robust estimates of the IC_50_s for individual isolates.

### Assessments of parasite clearance.

Parasite clearance metrics were assessed using the Worldwide Antimalarial Resistance Network parasite clearance estimator (23). Other efficacy endpoints are described in the supplemental text.

### Pharmacokinetic modeling.

Pharmacokinetics were initially described by noncompartmental analysis using Phoenix WinNonlin (version 6.2) software (Certara, Princeton, NJ, USA). PK-PD characteristics were then evaluated with nonlinear mixed-effects modeling in the software NONMEM (version 7.3; ICON Development Solutions, Ellicott City, MD, USA). The most appropriate model for PK characterization was developed by evaluating different absorption, disposition, and covariate models as well as random-effects components (see the supplemental text for details) (24, 25). Individual PK parameters from the final model were imputed into the PK-PD model to drive the cipargamin-dependent parasite-clearing effect.

### Pharmacodynamic modeling.

Parasite densities (determined by microscopy and qPCR) were transformed to total parasite burdens assuming 80 ml whole blood/kg of body weight. In P. falciparum malaria, as the density of asexual parasites declines, gametocytemia rises, because mature gametocytes are released from sequestration (26). DNA density estimates do not discriminate between asexual dividing, refractory, and sexual parasites. To reduce confounding by gametocytemia, the qPCR DNA density estimates were censored when sexual parasite densities (measured by the use of Pfs25 mRNA) reached >10% of the total measurement (assuming a conservative estimate of 10 mRNA copies per cell in the Pfs25 assay). Recrudescent infections were identified from rising qPCR DNA levels and were confirmed by rising ring-stage mRNA levels together with unchanged or falling Pfs25 transcript numbers; in some patients, this was confirmed by the appearance of asymptomatic microscopy-detectable asexual parasites. There was a good correlation between uncensored PCR and microscopy measurements (data not shown), and these data were therefore pooled for PK-PD modeling. Different structural PK-PD models were evaluated to characterize the relationship between drug exposure and parasite killing (see the supplemental text). The MICs were taken directly from the individually predicted profiles in patients with recrudescence.

### Statistical analysis.

The projected sample size was based on operational rather than statistical considerations (see the supplemental text). Parasite clearance and fever clearance times were analyzed using the Kaplan-Meier method (see the supplemental text). The relationship between cipargamin exposure, defined as AUC_0–24_ or *C*_max_, and PC_1/2_ was modeled with a nonlinear regression model, which is illustrated as follows for AUC_0–24_: PC_1/2_ = *a + (b − a) × e^−k × AUC_0–24_^, where a* is the asymptotic maximal effect of cipargamin exposure on the PC_1/2_, *b* is the longest PC_1/2_, and *k* is the rate of decrease in PC_1/2_.

## Supplementary Material

Supplemental material
