# Supplementary material for: Estimation of the In Vivo MIC of Cipargamin in Uncomplicated Plasmodium falciparum Malaria
Source: Antimicrob Agents Chemother. 2017 Jan 24;61(2):e01940-16. doi: 10.1128/AAC.01940-16 (PMC5278730; doi:10.1128/AAC.01940-16)
Supplement: Supplemental material [file supp_61_2_e01940-16__index.html]

Estimation of the In Vivo MIC of Cipargamin in Uncomplicated Plasmodium falciparum Malaria — Supplemental material 

# Estimation of the *In Vivo* MIC of Cipargamin in Uncomplicated Plasmodium falciparum Malaria

## Supplemental material

- Supplemental file 1 -

  Supplemental text, Figures S1 to S4, and Tables S1 to S3

  PDF, 703K
