# Supplementary material for: Estimation of the In Vivo MIC of Cipargamin in Uncomplicated Plasmodium falciparum Malaria
Source: Antimicrob Agents Chemother. 2017 Jan 24;61(2):e01940-16. doi: 10.1128/AAC.01940-16 (PMC5278730; doi:10.1128/AAC.01940-16)
Supplement: Supplemental material [file AAC.01940-16_zac002175877s1.pdf]

Supplemental material:

Supplementary methods

Exclusion criteria

Patients fulfilling any of the following criteria were not eligible for inclusion in the study:

- signs and symptoms of severe malaria according to World Health Organization (WHO) 2010 criteria
- infection with more than one species of malaria parasite
- use of other investigational drugs within 30 days or within five half-lives of enrollment, whichever was longer
- history of antimalarial use within 2 months of screening
- use of any antibiotics with antimalarial activity or other prohibited medication within 14 days of screening
- history of hypersensitivity to the study drug or to drugs of similar chemical classes
- long QT syndrome or QTc using Fridericia's formula >430 milliseconds for males and >450 milliseconds for females
- history of malignancy of any organ system (other than localized basal cell carcinoma of the skin), treated or untreated, within the past 5 years, regardless of whether there was evidence of local recurrence or metastases
- pregnant or lactating women (pregnancy confirmed by a positive hCG laboratory test)

- women of child-bearing potential, defined as all women physiologically capable of becoming pregnant, unless effective methods of contraception were used during dosing and for 30 days after administration of cipargamin

  Note: use of condom was required for sexually active males during intercourse while taking drug and for at least 30 days after stopping the study medication to not father a child in this period (use of condom required for vasectomized men in order to prevent delivery of the drug via seminal fluid)

- hemoglobin level <10 g/dL
- liver disease or injury as indicated by elevated liver tests such as alanine amino transferase or aspartate amino transferase >2 times the upper limit of normal (ULN)
- renal dysfunction as indicated by serum creatinine >2 × ULN in the absence of dehydration; in case of dehydration, serum creatinine should be <2 × ULN after oral or parental rehydration
- immunocompromised patients (including human immunodeficiency virus [HIV] infection) or receiving immunosuppressive therapy at the time of enrollment (HIV testing not required)
- known history of hepatitis B or C (testing not required)
- history of drug or alcohol abuse within one month prior to dosing, or clinical evidence of such abuse, febrile condition due to diseases other than malaria (e.g. acute lower respiratory tract infection)
- known underlying chronic or severe disease (e.g. cardiac, hepatic, renal, gastrointestinal, neurologic, or psychiatric disease), or any condition precluding enrollment into this study according to the investigator

- severe vomiting defined as >3 times during the previous 24 hours or inability to tolerate oral medication; severe diarrhea defined as ≥3 watery stools during the previous 24 hours
- severe malnutrition defined by a body mass index (BMI) <18.5 kg/m^2 or unintentional loss of body weight ≥10% with evidence of suboptimal intake resulting in loss of subcutaneous fat and/or severe muscle wasting
- active tuberculosis or history of taking anti-tuberculosis medications within 24 months prior to screening
- use of medications with known phototoxicity within 14 days of screening (e.g. tetracyclines, phenothiazines, chlorothiazide, sulfisoxazole, sulfanilamide, griseofulvin, tolbutamide)
- photodermatitis/increased sensitivity to sun, history of photosensitivity or porphyrias
- or any of the following skin diseases: systemic lupus erythematosus, dermatitis or psoriasis.

Criteria for standard of care treatment

Patients were given standard-of-care medication if any of the following criteria was met: return of fever or other symptoms of malaria, rise in parasitemia based on microscopy >100/µL at 72 hours post-dose (standard treatment was given due to clinical decline or treatment failure), presence of any parasitemia after completing 7 days of the study; three consecutive rising qPCR parasitemia with each step increased by >0.5 $\log_{10}$ until parasitemia reached 100/µL, or two consecutive rising qPCR parasitemia with at least one step increased by >1 $\log_{10}$.

Sample size considerations

A total of approximately 48 patients were to be recruited, eight patients per dose. This sample size was not based on statistical considerations, but rather chosen so that the study duration would be around 6–9 months based on expected recruitment rate, and was predicted to provide approximately 22–32 MIC observations, assuming a low rate of ETFs.

Analysis of secondary variables

Time to blood film parasite clearance (PC) and time to fever clearance were analyzed with the Kaplan-Meier method. The calculation was done on subjects in the PD analysis set. Subjects who discontinued treatment prior to parasite clearance due to AEs or lack of initial response were considered as right-censored at that point in time. Additional analyses of data collected from qPCR and/or microscopy included: the parasitemia slope half-life, as described by Flegg et al. (2011)[1] and given by $T_{1/2} = \log_e(2)/K = 0.692/K$, where K is the clearance treatment to the count at 24, 48, and 72 hours; the proportion of patients with ≥99% reduction from the baseline asexual parasite count at 24 hours after initiation of study drug; time to 99%/95%/50% reduction of asexual parasites ($PC_{99}/PC_{95}/PC_{50}$); and derived log parasite reduction ratio or the base 10 logarithm ratio of the parasite count before treatment to the count at 24 hours (PRRo 24), 48 hours (PRRo 48), and 72 hours (PRRo 72).

Population pharmacokinetic-pharmacodynamic modeling

Observed plasma cipargamin concentration measurements and parasite density measurements were transformed into their natural logarithms and evaluated with nonlinear mixed-effects modeling in the software NONMEM v 7.3 (ICON Development Solutions, Ellicott City, MD, USA). Post-processing and automation was performed using Pearl-Speaks-NONMEM (Psn) v 3.5.3,[2,3] Xpose v 4,[4] and R v 2.15.1 (The R Foundation for Statistical Computing, Vienna,

Austria). The objective function value (OFV), proportional to -2 × log-likelihood of data, was used to discriminate between hierarchical models. A drop in OFV of more than 3.84 was considered a significant improvement in model fit with a P value less than 0.05 at one degree of freedom difference. The first-order conditional estimation method with interactions was used throughout modeling, except for models including a probabilistic evaluation (e.g. M3 method for handling data below the LLOQ)[5] where the Laplacian estimation method was used. Model performance was evaluated by goodness-of-fit diagnostics, simulation-based diagnostics (visual predictive checks), and bootstrap diagnostics.

Pharmacokinetic modeling

One- and two-compartment disposition models were evaluated to find the most appropriate structural model. First-order absorption, with and without lag-time, as well as a more flexible transit-compartment absorption[6] with a fixed number of transit compartments (i.e. 1–10 transit compartments) were considered to describe the absorption profile of cipargamin. Pharmacokinetic parameters were assumed to be log-normally distributed with a normally distributed inter-individual random variability (zero mean and variance ω^2). Relative bioavailability, fixed to unity for the population, was evaluated to allow inter-individual variability in the same parameter. Residual random variability, which is the difference between the individual model prediction and the measured observation, was modelled as an additive error on the logarithmically transformed observation (essentially an exponential error on an arithmetic scale).

Body weight (centered on the median weight of the population) was evaluated as a fixed allometric function on all clearance and volume parameters (exponent of 0.75 for clearance parameters and 1.0 for volume parameters, respectively).[7] Dose was evaluated as a linear covariate relationship on all pharmacokinetic parameters.

The final pharmacokinetic structural model and individual pharmacokinetic parameter estimates were imputed into the pharmacodynamic model to derive the drug-dependent killing of parasites.

Pharmacodynamic modeling

Observed parasitemia derived from qPCR or microscopy was modelled simultaneously, with no discrimination between the two, in order to maximize the amount of information available. However, censored qPCR measurements were omitted due to the uncertainty in these observations, while microscopy observations below the limit of quantification were modelled as categorical data using the previously published M3 method.[5] The PK–PD base model comprised of a homogenous parasite population (P) with a fixed parasite growth rate (K_{grow}) and a drug-dependent killing of parasites (K_{kill}) (Eq. 1 and Eq. 2).

$$\frac{dP}{dt} = P \times K_{grow} - P \times K_{kill} \qquad \text{Equation 1.}$$

$$K_{kill} = E_{max} \times \frac{C_P}{C_P + EC_{50}} \qquad \text{Equation 2.}$$

where E_{MAX} is the maximum parasite killing, C_P is the predicted plasma concentration of cipargamin and EC_{50} is the cipargamin concentration needed for half of the maximum effect.

Baseline parasitemia (P_{bas}) was estimated initially, allowing for inter-individual variability in the same parameter. This was later imputed directly into the model as a mean of the individually observed qPCR- and microscopy-based parasitemia but allowing for a residual error, for a more parsimonious model (i.e. minimize the number of parameters to be estimated). The hypothesis of coexisting drug insensitive (P_{ns}) and sensitive (P_s) parasite populations was evaluated by the addition of a hypothetical drug refractory compartment with non-dividing non-cleared parasites. The proportion of sensitive parasites (F_{sen}) was estimated at baseline (Eq. 3 and 4), and assumed to be unaffected by the drug. A first-order activation

constant (K_{act}) for non-sensitive parasites to become sensitive (Eq. 5) was assumed to account for patients being cured. The resulting overall parasite model is shown below (Eq. 3 to 6).

$$P_{ns_{(t=0)}} = P_{bas} \times (1 - F_{sen}) \qquad \text{Equation 3.}$$

$$P_{s_{(t=0)}} = P_{bas} \times F_{sen} \qquad \text{Equation 4.}$$

$$\frac{dP_{ns}}{dt} = -P_{ns} \times K_{act} \qquad \text{Equation 5.}$$

$$\frac{dP_S}{dt} = P_s \times K_{grow} - P_s \times K_{kill} + P_{ns} \times K_{act} \qquad \text{Equation 6.}$$

where the observed total parasitemia is the sum of sensitive and non-sensitive parasites. An arbitrary cut-off of 10 parasites was used to define cure. This was implemented in the pharmacodynamic model by fixing K_{grow} to zero when the total number of sensitive parasites fell below this value.

The implementation of a hypothetical effect compartment (i.e. indirect response model) was also evaluated to account for a potentially delayed response. An estimated first-order transfer of drug to and from the hypothetical effect compartment governed the appearance and elimination of drug in the effect compartment. The concentration of drug in the hypothetical effect compartment was used to modulate the parasite killing effect. The effect of dose (centered on the lowest dose group of 10 mg) on E_{max} was evaluated as a power relationship (Eq. 7). No other covariates were evaluated in this relatively small study.

$$E_{max,i} = E_{max,TV} \times \left(\frac{Dose}{10}\right)^{\theta} \qquad \text{Equation 7.}$$

where $E_{max,i}$ is the individually estimated maximum killing of parasite, $E_{max,TV}$ is the typical value for the population, Dose is the administered dose and θ is the estimated exponent of the covariate relationship.

Pharmacodynamic parameters were assumed to be log-normally distributed with a normally distributed inter-individual random variability (zero mean and variance ω^2). Residual random variability, which is the difference between the individual model prediction and the measured observation, was modelled as an additive error on the logarithmically transformed observation (essentially an exponential error on an arithmetic scale).

Supplementary results

Population pharmacokinetic-pharmacodynamic modeling

Pharmacokinetic modeling

A one-compartment disposition model described the pharmacokinetic properties of cipargamin well. An additional disposition compartment resulted in a significantly improved model fit ($P < 0.05$) but no improvement could be seen in simulation-based diagnostics. However, a two-compartment disposition model resulted in an unrealistically small central volume of distribution (0.26 L) and was therefore not carried forward. A flexible transit-compartment model was superior to a first-order absorption model with or without lag-time ($P < 0.05$). Three transit-compartments were found to be optimal to describe the absorption profile of cipargamin in this patient population. Body weight implemented as a fixed allometric function on clearance and volume parameters was superior to a covariate free model (ΔOFV = 3.96). Dose was evaluated as a linear covariate on all pharmacokinetic parameters and produced no significant improvements in model fit. Inter-individual variability on central volume of distribution was estimated close to zero and therefore fixed to zero. Individual concentration-time profiles showed an excellent fit to observed data and the pharmacokinetic model was deemed appropriate to be incorporated into the pharmacodynamic model.

Pharmacodynamic modeling

Parasite clearance was clearly biphasic and implementation of a hypothetical drug refractory sub-population resulted in a substantial improvement in model fit ($P < 0.05$). Other mechanisms for the biphasic pattern, such as two separate drug-dependent parasite elimination rates, cannot be excluded.

A shape factor (i.e. Hill coefficient) of the exposure–response model was not estimated due to the sparseness of the data. However, it is likely that the model would benefit by refining this relationship with more data available. A delayed-response model (i.e. an effect–compartment model) was also evaluated but resulted in an unstable model (minimization terminated). Malaria parasites are restricted to the red blood cells and so it is likely that the transfer of drug from whole blood to the effect site is rapid. However, a minor model mis-specification was seen in the first 5 hours after drug administration (Fig. S4) suggesting a slightly delayed parasite killing. Not estimating a delayed response should have minimal impact on the overall model performance. Dose was a significant covariate resulting in an increased maximum killing of parasites with increasing doses.

Inter-individual variability could be retained on maximum parasite killing, fraction of sensitive parasites and the activation rate of refractory parasites. The final pharmacodynamic model showed adequate model performance but more data are needed to define all parameters accurately.

Supplementary references

1. **Flegg JA, Guerin PJ, White NJ, Stepniewska K.** 2011. Standardizing the measurement of parasite clearance in falciparum malaria: the parasite clearance estimator. Malar J **10:**339.
2. **Lindbom L, Ribbing J, Jonsson EN.** 2004. Perl-speaks-NONMEM (PsN)—a Perl module for NONMEM related programming. Comput Methods Programs Biomed **75:**85–94.
3. **Lindbom L, Pihlgren P, Jonsson EN.** 2005. PsN-Toolkit—a collection of computer intensive statistical methods for non-linear mixed effect modeling using NONMEM. Comput Methods Programs Biomed **79:**241–257.
4. **Jonsson EN, Karlsson MO.** 1999. Xpose—an S-PLUS based population pharmacokinetic/pharmacodynamic model building aid for NONMEM. Comput Methods Programs Biomed **58:**51–64.
5. **Beal SL.** 2001. Ways to fit a PK model with some data below the quantification limit. J Pharmacokinet Pharmacodyn **28:**481–504.
6. **Savic RM, Jonker DM, Kerbusch T, Karlsson MO.** 2007. Implementation of a transit compartment model for describing drug absorption in pharmacokinetic studies. J Pharmacokinet Pharmacodyn **34:**711–726.
7. **Holford NH.** 1996. A size standard for pharmacokinetics. Clin Pharmacokinet **30:**329–332.

Supplementary figures

FIG S1. Patient disposition by dose group

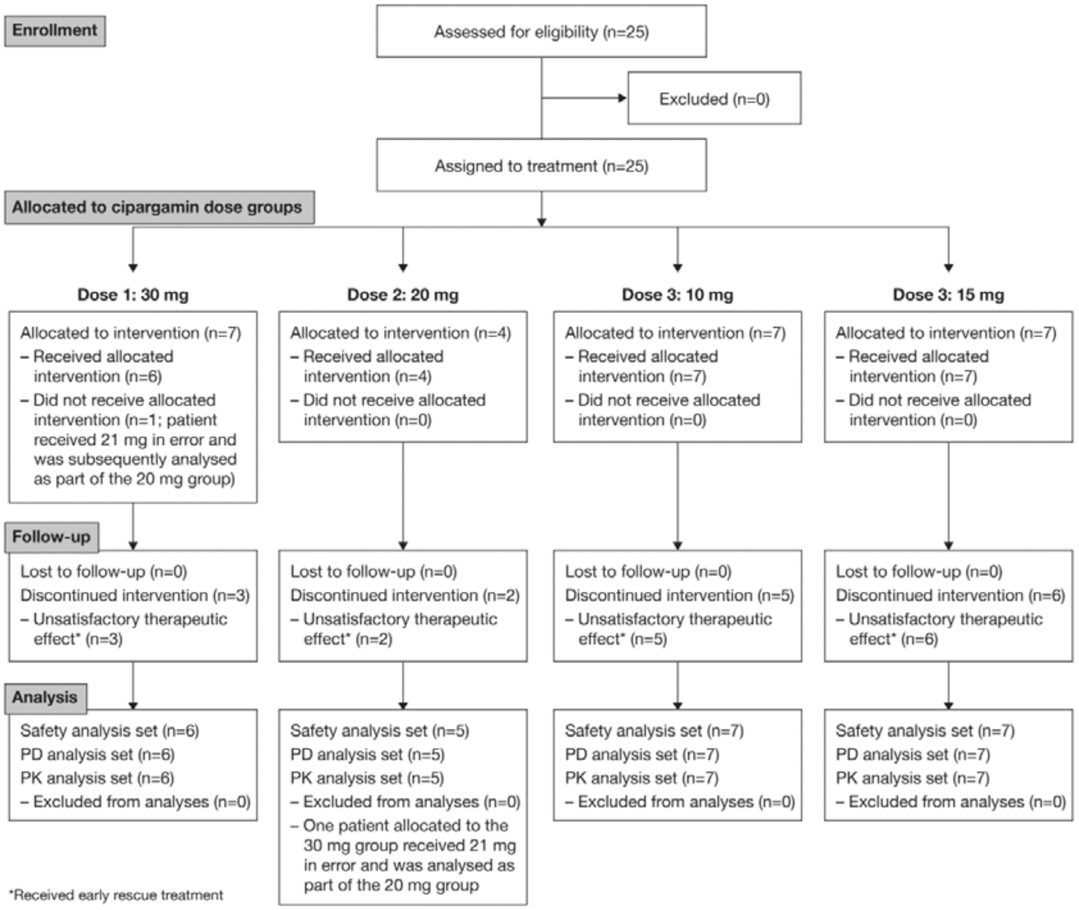

FIG S2. Mean arithmetic (upper panel) and semi-logarithmic (lower panel) plasma concentration–time profiles of cipargamin following single oral administration in patients with uncomplicated *P. falciparum* malaria

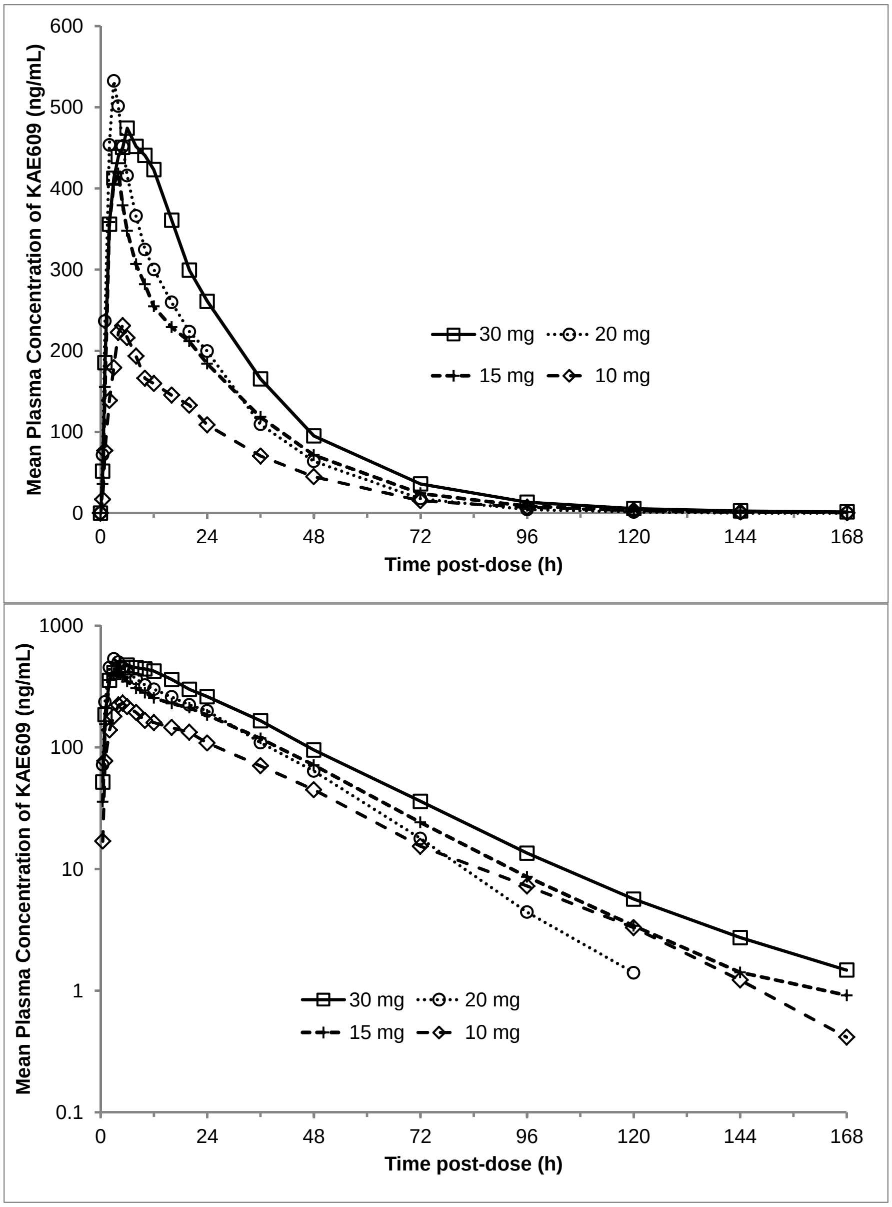

FIG S3. Goodness-of-fit diagnostics of the final model describing cipargamin pharmacokinetics (A) and pharmacodynamics (B) in patients with uncomplicated *P. falciparum* malaria

Observations, population predictions, and individual predictions were transformed into their logarithms (base 10).

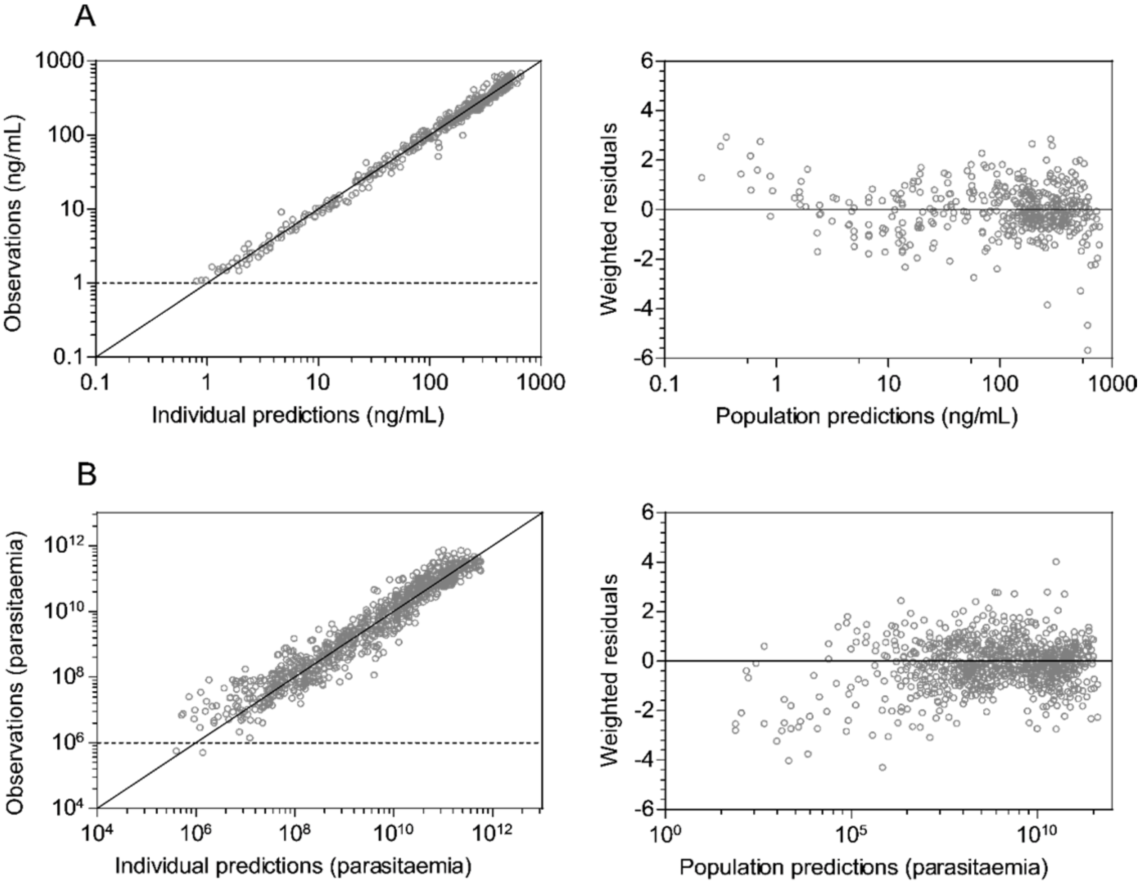

FIG S4. Visual predictive checks of the final model describing cipargamin pharmacokinetics (A) and pharmacodynamics (B) in patients with uncomplicated *P. falciparum* malaria

Open circles represent the observations. Solid black lines represent the 5th, 50th, and 95th percentiles of the observations. Grey areas represent the 95% confidence intervals of the simulated 5th, 50th, and 95th percentiles from 2,000 simulations. The insets show a visual predictive check for the first day of treatment.

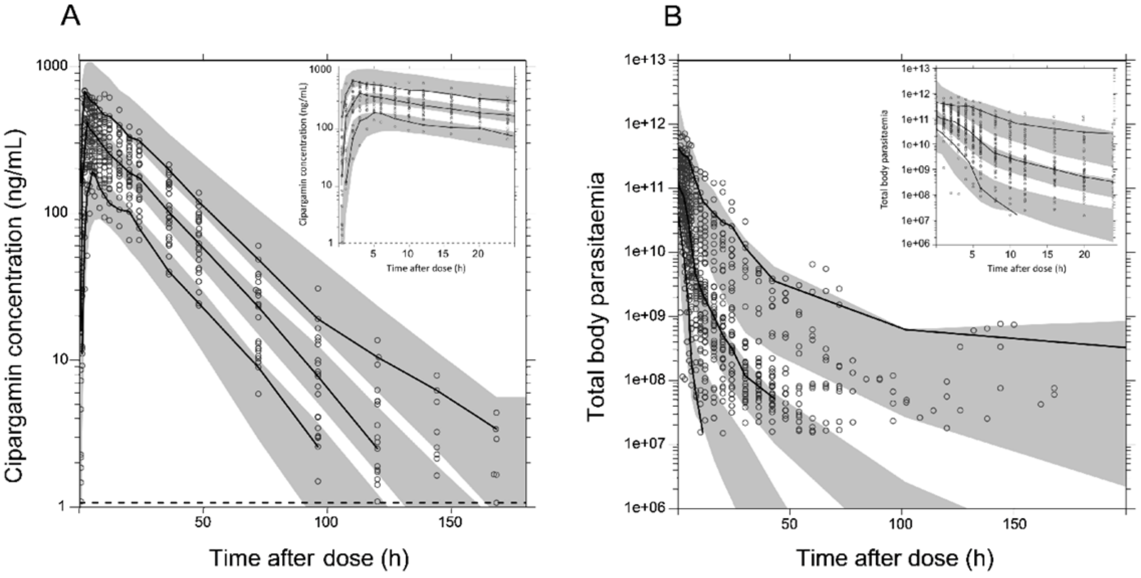

Supplementary tables

TABLE S1. Efficacy data by cipargamin dose (PD analysis set)

Variable		Dose			
		**30 mg** *n* = 6	**20 mg** *n* = 5	**10 mg** *n* = 7	**15 mg** *n* = 7
Median time to microscopic parasite clearance (h)		30.0	54.0	54.0	60.0
Median time to fever clearance (h)		6.3	11.9	15.8	11.8
28-day cure rate (%) (PCR-corrected)[a]		50	60	29	14
Mean PRRo 24 ± SD (range)		3.439 ± 0.5858 (2.41–3.98)	3.129 ± 0.6986 (2.26–4.01)	1.826 ± 0.9171 (0.68–3.08)	2.201 ± 0.7209 (1.39–3.20)
Mean PRRo 48 ± SD (range)		3.717 ± 0.4522 (3.13–4.33)	3.690 ± 0.2739 (3.42–4.01)	3.083 ± 0.9628 (1.54–4.21)	3.494 ± 0.6429 (2.72–4.26)
Mean PRRo 72 ± SD (range)		3.754 ± 0.4143 (3.13–4.33)	3.880 ± 0.3675 (3.43–4.40)	3.302 ± 0.6981 (2.03–4.21)	3.815 ± 0.4670 (3.01–4.26)
PC_{99} (h)		10.02	6.00	24.08	20.00
PC_{95} (h)		8.00	5.00	12.00	10.00
PC_{50} (h)		5.50	3.00	5.00	4.00
Mean percent reduction in asexual parasitemia from baseline	At 24 hours post-dose (%)	99.9	99.8	94.1	98.5
	At 48 hours post-dose (%)	100.0	100.0	99.4	99.9
Proportion of patients with gametocytemia[b]	Before treatment (%)	0	0	14	0
	After treatment (%)	83	80	57	86
Proportion of patients aparasitemic by microscopy	At 24 hours post-dose (%)	50	20	0	0
	At 48 hours post-dose (%)	83	40	29	43
	At 72 hours post-dose (%)	100	80	57	71
Proportion of patients with ETF (%)		0	20	43	0

[a] Uncorrected and PCR-corrected cure rates were identical in all four cases. Only Day 28 results are presented here as they were identical to those at Day 42; [b]Gametocytemia was analyzed by microscopy only.

ETF, early treatment failure; min, minimum value; max, maximum value; $PC_{99, 95, 50}$, time to 99%, 95%, and 50% reduction of parasite respectively; PRRo, derived log parasite reduction ratio or the base 10 logarithm ratio of the parasite count before treatment to the count at 24 hours (PRRo 24), 48 hours (PRRo 48), and 72 hours (PRRo 72); SD, standard deviation.

Secondary endpoint data are presented in the table above. The lowest median time to microscopy parasite clearance and time to fever clearance were both in the 30 mg group (30.0 and 6.3 hours, respectively). Cure rates at 28 days were 50%, 60%, 29% and 14% for the 30, 20, 10 and 15 mg groups, respectively. One patient (in the 10 mg group) had gametocytemia at baseline. Nineteen patients developed gametocytemia during the study. At all points during the study, gametocyte count was higher in the 30 mg and 15 mg groups. All but two patients ($n = 1$ in both 10 and 15 mg groups) were gametocyte free by day 42. All patients in the 30 mg group were aparasitemic by microscopy at 72 hours post-dose, compared with 80%, 57% and 71% of patients in the 20, 10 and 15 mg groups, respectively. No patients in the 30 and 15 mg groups had early treatment failures; this occurred in 3 (43%) and 1 (20%) patients with 10 and 20 mg, respectively.

TABLE S2. Incidence of adverse events

	**Dose 1: 30 mg *n* = 6**	**Dose 2: 20 mg *n* = 5**	**Dose 3: 10 mg *n* = 7**	**Dose 4: 15 mg *n* = 7**	**Total *n* = 25**
Any AE	4 (67)	3 (60)	7 (100)	5 (71)	19 (76)
Blood and lymphatic system disorders	0	0	1 (14)	0	1 (4)
Anemia	0	0	1 (14)	0	1 (4)
Gastrointestinal disorders	1 (17)	1 (20)	2 (29)	1 (14)	5 (20)
Nausea	1 (17)	0	1 (14)	0	2 (8)
Abdominal pain upper	0	0	1 (14)	0	1 (4)
Abnormal feces	0	1 (20)	0	0	1 (4)
Diarrhea	0	0	0	1 (14)	1 (4)
Hepatobiliary disorders	0	1 (20)	3 (43)	1 (14)	5 (20)
Hyperbilirubinemia	0	1 (20)	3 (43)	1 (14)	5 (20)
Investigations	1 (17)	2 (40)	3 (43)	4 (57)	10 (40)
Blood alkaline phosphatase increased	1 (17)	2 (40)	3 (43)	4 (57)	10 (40)
Alanine aminotransferase increased	1 (17)	0	0	0	1 (4)
Musculoskeletal and connective tissue disorders	1 (17)	0	0	0	1 (4)
Myalgia	1 (17)	0	0	0	1 (4)
Nervous system disorders	1 (17)	1 (20)	0	0	2 (8)
Headache	1 (17)	1 (20)	0	0	2 (8)
Respiratory, thoracic and mediastinal disorders	0	1 (20)	0	0	1 (4)
Cough	0	1 (20)	0	0	1 (4)
Skin and subcutaneous tissue disorders	0	0	0	1 (14)	1 (4)
Rash vesicular	0	0	0	1 (14)	1 (4)
Vascular disorders	1 (17)	0	0	0	1 (4)
Hypertensive crisis	1 (17)	0	0	0	1 (4)

All values are *n* (%, rounded to the nearest integer)

TABLE S3. Summary of pharmacokinetic parameters from non-compartmental analysis of cipargamin plasma concentration–time profile following single oral administration to patients with uncomplicated *P. falciparum* malaria

**Parameter**	**30 mg** *n* **= 6**	**20 mg** *n* **= 5**	**15 mg** *n* **= 7**	**10 mg** *n* **= 7**
C_{max} (ng/mL)	557 ± 158 [28.4]	562 ± 98 [17.5]	462 ± 108 [23.4]	253 ± 80 [31.7]
Tmax (h)	3.51 (2–10)	3.00 (2–4)	3.00 (2–4)	4.02 (2–6)
$AUC_{0–24h}$ (µg.h/mL)	8.75 ± 2.12 [24.2]	7.39 ± 1.25 [16.9]	6.28 ± 1.59 [25.2]	3.69 ± 0.892 [24.2]
AUC_{inf} (µg.h/mL)	15.5 ± 4.11 [26.6]	11.6 ± 3.16 [27.1]	11.0 ± 3.20 [29.0]	6.6 ± 1.83 [27.7]
AUC_{last} (µg.h/mL)	15.4 ± 4.10 [26.6]	10.9 ± 3.18 [29.1]	11.0 ± 3.19 [29.0]	7.37 ± 1.61 [21.8]
$T_{1/2}$ (h)	19.4 ± 8.05 [41.5]	14.7 ± 1.72 [11.7]	17.6 ± 7.09 [40.4]	18.5 ± 3.45 [18.6]
CL/F (L/h)	2.13 ± 0.872 [41.0]	1.84 ± 0.486 [26.4]	1.51 ± 0.627 [41.6]	1.63 ± 0.506 [31.1]
Vz/F (L)	55.4 ± 18.9 [34.1]	39.2 ± 12.6 [32.0]	36.7 ± 16.0 [43.7]	42.7 ± 12.1 [28.3]

All values are mean ± SD [CV%] except for Tmax which is median (range). Coefficient of variation (in %) is given in square brackets.

C_{max}, maximum (peak) plasma drug concentration after drug administration; Tmax, time to reach peak or maximum plasma concentration following drug administration; $AUC_{0–24h}$, area under the plasma concentration–time curve during from time zero to 24 hours post-dose; AUC_{inf}, area under the plasma concentration–time curve from time zero to infinity; AUC_{last}, area under the plasma concentration–time curve from time zero to the time of the last quantifiable concentration; $T_{1/2}$, terminal elimination half-life; CL/F, apparent systemic (or total body) clearance from plasma following extravascular administration; Vz/F, apparent

volume of distribution during the terminal elimination phase following extravascular administration.
